# BETi induction of mitotic catastrophe: towing the LIN9

**DOI:** 10.18632/oncoscience.372

**Published:** 2017-10-23

**Authors:** Sylvia S. Gayle, Jennifer M. Sahni, Ruth A. Keri

**Affiliations:** Department of Pharmacology, Case Western Reserve University, Cleveland, OH 44106, USA; Department of Genetics and Genome Sciences, Case Western Reserve University, Cleveland, OH 44106, USA; Department of General Medical Sciences-Oncology, Case Western Reserve University, Cleveland, OH 44106, USA

**Keywords:** LIN9, mitotic catastrophe, BET inhibitor, breast cancer, oncogene

**Figure 1:**
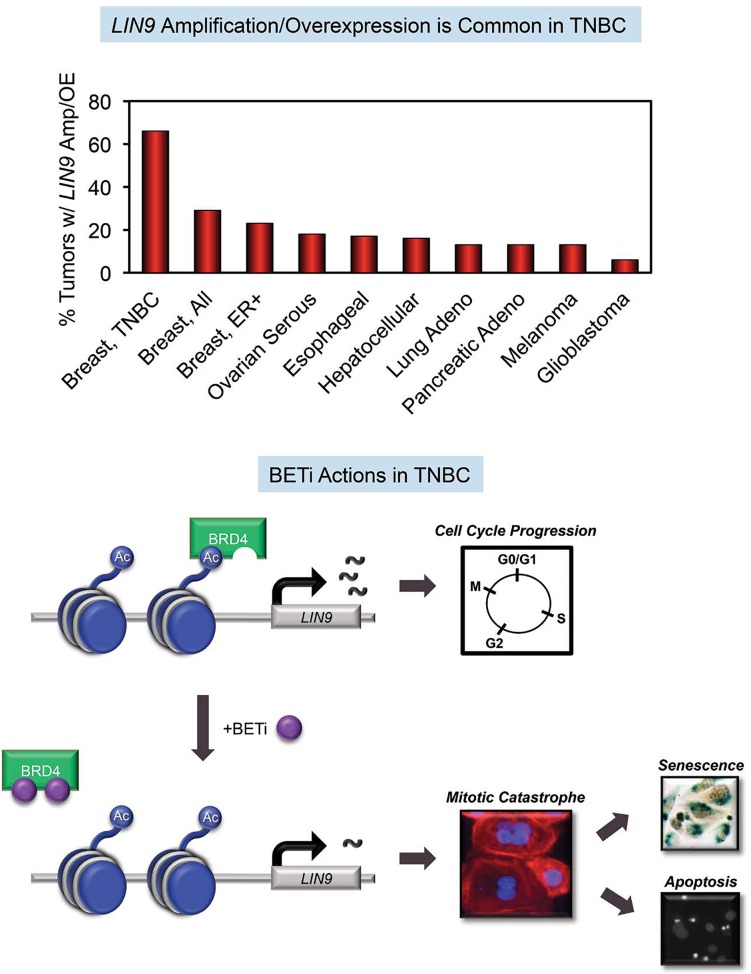
*LIN9* overexpression is a vulnerability that can be targeted with BETi to induce mitotic catastrophe in TNBC

Targeting proliferating cells has been a mainstay of chemotherapy, yet this approach is associated with widespread toxicity. Identifying factors that control cell cycle progression in a tumor-selective manner should provide avenues for targeted therapy that are associated with less systemic side effects. The MuvB complex is composed of five subunits (LIN9, LIN37, LIN52, LIN54, and RBBP4) and, together with FOXM1 and B-MYB, is critical for proper cell cycle progression and mitosis [1]. LIN9 acts as a tumor suppressor in some cancers [2]. However, in breast cancer it is a component of the metastasis-predicting Mammaprint gene signature [3], despite a lack of understanding of its role in this disease. We recently discovered that LIN9 represents a mitotic vulnerability in triple-negative breast cancers (TNBCs) [4]. Interrogation of publically available datasets revealed about two-thirds of TNBC tumors and one-quarter of all breast cancers have amplified or overexpressed *LIN9* (Fig 1A). Moreover, high *LIN9* expression is associated with poor outcomes. These data point to LIN9 as a potential therapeutic target in breast cancer, particularly in TNBC which is highly aggressive and currently lacks effective targeted therapies.

Our studies revealed that LIN9 is an important mediator of the effects of inhibitors of the Bromodomain and Extraterminal (BETi) family of epigenetic readers in TNBC [4]. BET proteins (BRD2, BRD3, BRD4, and BRDT) have two tandem bromodomains that bind to acetylated lysines in histone tails associated with chromatin. They facilitate the activation of transcription, and in cancer, BRD4 preferentially localizes to regulatory sequences in oncogenes, ensuring their sustained activation [5, 6]. BETi, such as the small molecule JQ1, target the epigenome by competitively binding to the bromodomains of BET proteins, thereby blocking their ability to interact with acetylated histones and thus preventing oncogene transcription (Fig 1B) [6]. We and others have reported that BETi effectively suppress growth of TNBC cells *in vitro* and *in vivo* and reduce liver metastasis while having no impact on the normal mammary gland [7]. Extended exposure to BETi induces apoptosis or senescence in a cell line-dependent manner, and both of these outcomes are preceded by the appearance of multinucleated cells and downregulation of cell cycle and mitosis genes [4, 7].

Within six hours, BETi suppress expression of LIN9 as well as four additional master mitosis transcriptional regulators, FOXM1, E2F2, E2F8, and B-MYB (encoded by *MYBL2*), by reducing BRD4 binding to the promoter regions of their respective genes [4]. By individually silencing each transcription factor using siRNAs, we found that the loss of *LIN9* induced similar responses as BETi such as altered expression of cell cycle genes and induction of multinucleation. Furthermore, *in silico* analyses revealed that genes whose expression correlated with *LIN9* and/or had a LIN9 binding site were more susceptible to BETi suppression. These data indicated that the *LIN9* gene is a principal target of BET proteins that is necessary for maintaining mitotic progression. However, suppression of the other four transcription factors also induced some level of multinucleation and suppression of cell cycle genes, suggesting that while *LIN9* may drive the response of TNBCs to BETi, *FOXM1*, *E2F2*, *E2F8*, and *MYBL2* may also contribute.

The anti-cancer activity and preferential targeting of cancer cells by BETi are thought to be the result of super- enhancer (SE) disassembly at oncogenes [5]. However, when we generated a genome-wide SE map in the MDA- MB-231 TNBC cell line using H3K27Ac ChIP-seq, we found neither *LIN9* nor the four other mitosis-regulating transcription factors had putative SEs at their promoters [4]. Rather, multiple lines of evidence, including increased multinucleation, extended mitotic timing, and the induction of senescence or mitosis-associated apoptosis, indicated that downregulation of *LIN9* by BETi induces mitotic catastrophe. Cancer cells are more sensitive to mitotic catastrophe than normal cells [8], hence our findings suggest a novel mechanism for the selectivity of BETi in cancer cells.

Together, our studies identify LIN9 as a novel therapeutic target in TNBC that can be suppressed using BETi and suggest that LIN9 expression may define a subset of cancers that are particularly vulnerable to these drugs. Downregulation of LIN9 by BETi induces apoptosis and senescence in TNBC cells by initiating mitotic catastrophe. The identification of mitotic catastrophe as a mechanism of action of BETi should facilitate identifying patients who would be more likely to respond to BETi (*i.e.,* those with LIN9 overexpression), predicting potential toxicities, and designing rational drug combination therapies. Future studies are needed to elucidate the full function of LIN9 in TNBC and to determine if it acts as an oncogene in breast and other cancers. If overexpression of LIN9 is found to be a vulnerability in additional cancers, the response of these cancers to BETi should be assessed. Lastly, it will be important to determine if other agents that elicit similar effects as BETi, such as CDK7 inhibitors and dual kinase/BET inhibitors, also require suppression of LIN9 for their activity in TNBC.
